# The Impact of Social Media Interventions on Weight Reduction and Physical Activity Improvement Among Healthy Adults: Systematic Review

**DOI:** 10.2196/38429

**Published:** 2023-03-16

**Authors:** Wa'ed Shiyab, Elizabeth Halcomb, Kaye Rolls, Caleb Ferguson

**Affiliations:** 1 School of Nursing, Faculty of Science, Medicine & Health University of Wollongong Wollongong Australia

**Keywords:** social media, physical activity, overweight, lifestyle risk factors

## Abstract

**Background:**

A sedentary lifestyle and being overweight or obese are well-established cardiovascular risk factors and contribute substantially to the global burden of disease. Changing such behavior is complex and requires support. Social media interventions show promise in supporting health behavior change, but their impact is unclear. Moreover, previous reviews have reported contradictory evidence regarding the relationship between engagement with social media interventions and the efficacy of these interventions.

**Objective:**

This review aimed to critically synthesize available evidence regarding the impact of social media interventions on physical activity and weight among healthy adults. In addition, this review examined the effect of engagement with social media interventions on their efficacy.

**Methods:**

CINAHL and MEDLINE were searched for relevant randomized trials that were conducted to investigate the impact of social media interventions on weight and physical activity and were published between 2011 and 2021 in the English language. Studies were included if the intervention used social media tools that provided explicit interactions between the participants. Studies were excluded if the intervention was passively delivered through an app website or if the participants had a known chronic disease. Eligible studies were appraised for quality and synthesized using narrative synthesis.

**Results:**

A total of 17 papers reporting 16 studies from 4 countries, with 7372 participants, were identified. Overall, 56% (9/16) of studies explored the effect of social media interventions on physical activity; 38% (6/16) of studies investigated weight reduction; and 6% (1/16) of studies assessed the effect on both physical activity and weight reduction. Evidence of the effects of social media interventions on physical activity and weight loss was mixed across the included studies. There were no standard metrics for measuring engagement with social media, and the relationship between participant engagement with the intervention and subsequent behavior change was also mixed. Although 35% (6/16) of studies reported that engagement was not a predictor of behavior change, engagement with social media interventions was found to be related to behavior change in 29% (5/16) of studies.

**Conclusions:**

Despite the promise of social media interventions, evidence regarding their effectiveness is mixed. Further robust studies are needed to elucidate the components of social media interventions that lead to successful behavior change. Furthermore, the effect of engagement with social media interventions on behavior change needs to be clearly understood.

**Trial Registration:**

PROSPERO International Prospective Register of Systematic Reviews CRD42022311430; https://www.crd.york.ac.uk/prospero/display_record.php?RecordID=311430

## Introduction

### Background

Regular physical activity and weight management are well-established strategies for cardiovascular risk reduction. Maintaining a healthy lifestyle has been shown to increase the number of years lived free from chronic diseases and improve overall physical and emotional well-being [1,2]. Conversely, being overweight or obese is associated with chronic diseases and poor quality of life [3]. Therefore, it is strongly recommended that adults maintain a normal BMI (18.5-24.9 kg/m^2^) through a healthy balanced diet and regular physical activity (at least 150 minutes of moderate intensity or 75 minutes of vigorous physical activity per week) [4,5]. However, sedentary lifestyles and being overweight or obese are becoming increasingly common. Worldwide, one-quarter of adults are inactive, and approximately 52% of adults are affected by overweight or obesity [6,7].

Various interventions have been used to manage weight and promote physical activity with variable effects [8]. Social media can be a powerful and cost-effective platform for delivering behavior modification interventions [9]. Social media is defined as “internet-based channels that allow users to opportunistically interact and selectively self-present, either in real-time or asynchronously, with both broad and narrow audiences who derive value from user-generated content and the perception of interaction with others” [10]. Social media interventions are delivered using social media platforms (eg, Facebook and Twitter) to improve or maintain healthy behaviors [11]. Social media use is ubiquitous, with 80% of Australians using the internet to access social media and 72% of Americans using at least 1 type of social media [12,13]. In Europe, 85% of the population uses social media [14].

Engagement is an important factor in determining the effectiveness of social media interventions [15]. However, sustaining a person’s engagement with digital interventions is often challenging [16]. To improve the efficacy of social media interventions and boost their use in “real life”, it is crucial to understand how individuals interact with them [17]. Engagement can be measured in various ways, including the time spent on social media, number of likes or posts, or number of log-ins. However, no standard definition or measurement of engagement has been reported in the literature [15].

### Objective

Previous reviews have assessed the effects of social media interventions on various health behaviors [18-21]. These reviews have some limitations as they did not focus on specific age groups (eg, adults) and included studies of low methodological quality and heterogeneous intervention features and methodological approaches [19-21]. In addition, the evidence from these reviews regarding the efficacy of social media interventions in promoting healthy behaviors is inconclusive. Furthermore, none of the previous reviews examined how participants’ interactions and engagement with social media interventions affected their efficacy. Therefore, this review was undertaken to provide a critical synthesis of randomized controlled trials that reported the efficacy of social media interventions on physical activity and weight loss among healthy adults.

## Methods

### Research Design

The PRISMA (Preferred Reporting Items for Systematic Reviews and Meta-Analyses) guidelines [22] were used to guide the reporting of this systematic review. The review was registered in PROSPERO in February 2022 (registration ID: CRD42022311430).

### Search Strategy

**Figure 1:**
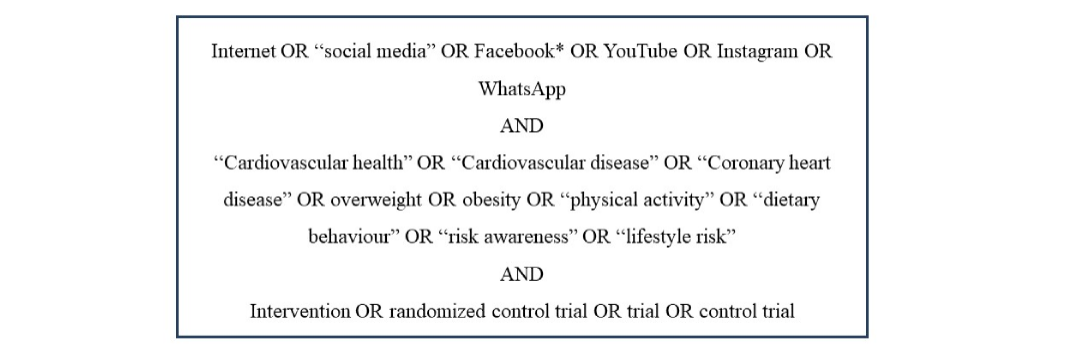
Search strategy.

MEDLINE and CINAHL were searched for relevant randomized controlled trials that were conducted to assess the effectiveness of social media interventions on weight loss and physical activity that were published between 2011 and 2021 (Figure 1). This period was selected to reflect recent trends in the increasing use of social media in the community [23]. Keywords were selected based on consultation with a university librarian. Social media types were based on common use among adults at the time of the review.

### Eligibility Criteria

Studies were included if they were randomized trials published in the English language and focused on social media interventions to enhance at least one of the following behaviors: physical activity and weight reduction in healthy adults. The outcomes were either self-reported or objectively measured. In this review, a healthy adult was defined as a person with no documented chronic conditions that were the focus of the intervention. Studies were included if the intervention used social media tools (as a single intervention or one of the components) that provided explicit interaction between participants. Studies were excluded if the intervention was passively delivered through an app website or if the participants had a known chronic disease. In addition, studies that evaluated the cost-effectiveness of or engagement with social media interventions as the primary outcome were excluded.

### Study Screening and Selection

**Figure 2:**
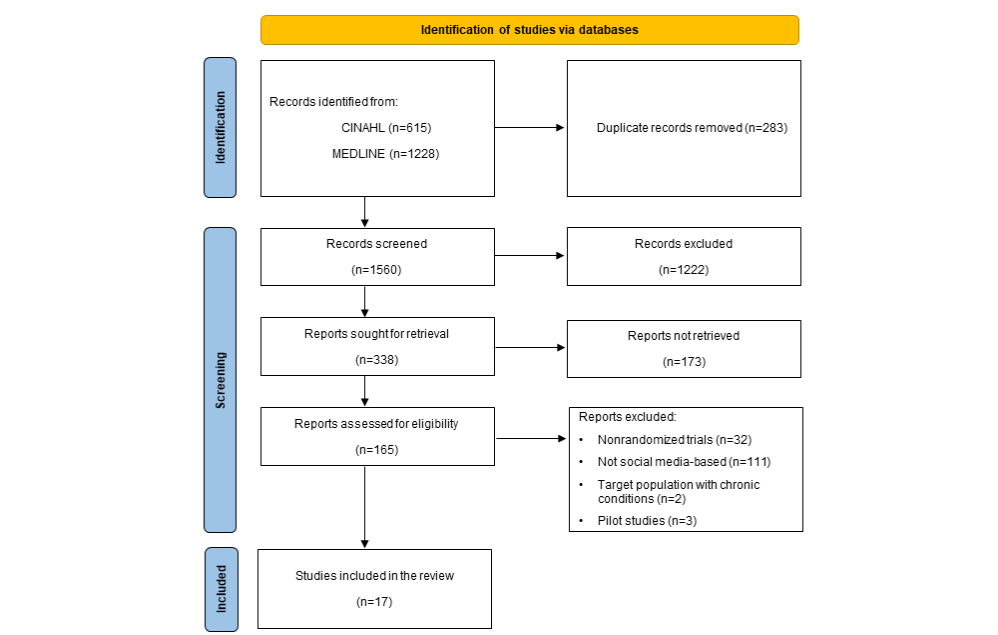
PRISMA (Preferred Reporting Items for Systematic Reviews and Meta-Analyses) flowchart.

The search results were exported to EndNote (version 20; Clarivate Analytics) [24] where duplicates were removed (Figure 2). Titles and abstracts were screened by 1 reviewer (WS), and studies that did not meet the inclusion criteria were excluded. A total of 1222 (78.3%) papers were removed after title screening 1560 records, and 173 (51.2%) papers were removed from 338 reports after reviewing the abstracts. Two authors (WS and EH) reviewed the full text of the remaining 165 papers. Uncertainty regarding the inclusion of studies was resolved through a discussion between 3 reviewers (WS, EH, and KR). Of these 165 papers, 17 (10.3%) met the inclusion criteria and were included in this review.

### Data Extraction and Quality Assessment

**Figure 3:**
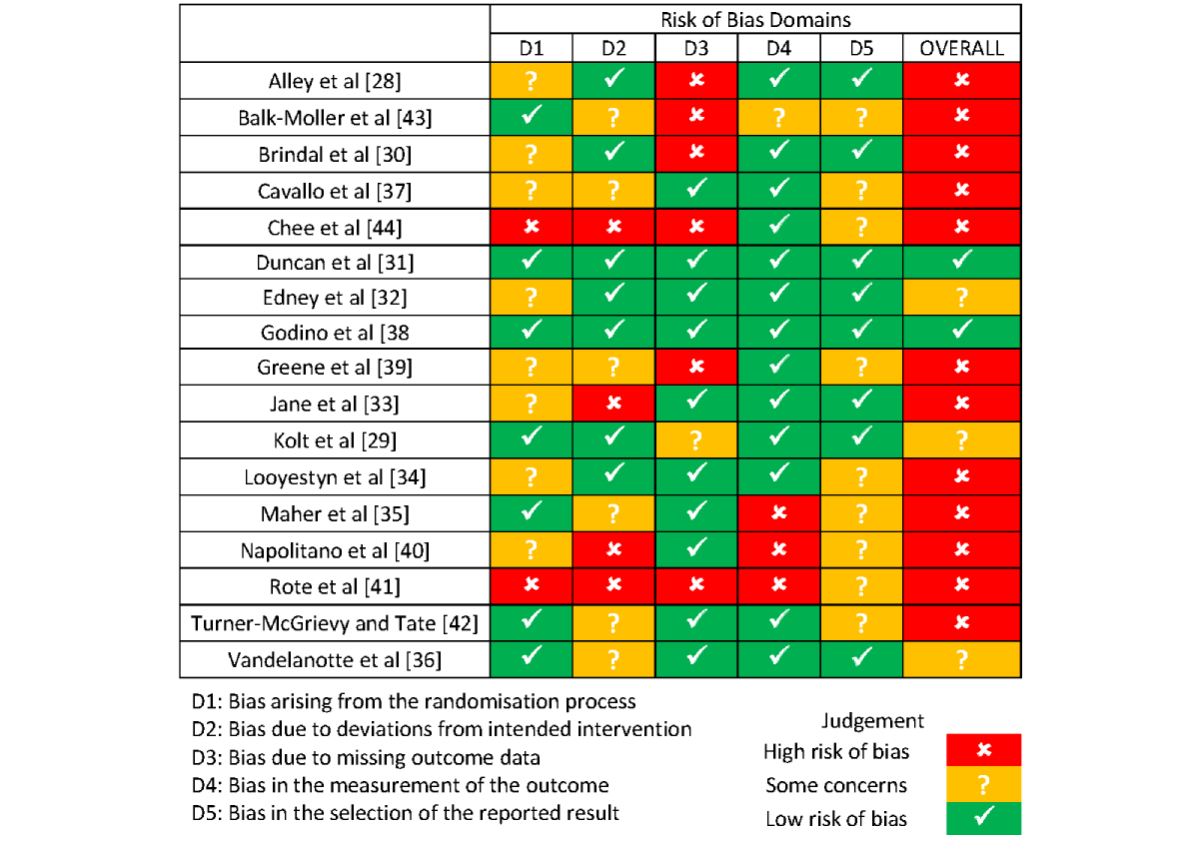
Quality appraisal.

Data on the characteristics of the included studies, including the year of publication, country, intervention, outcome measures, and major findings, were extracted by 1 reviewer (WS) and reviewed for completeness by 3 authors (WS, EH, and KR). These data were extracted into a standardized summary table using a combination of free text and predetermined categories. Three reviewers (WS, KR, and CF) independently assessed the quality of each included study using the revised Cochrane risk-of-bias tool for randomized trials (ROB2; The Cochrane Collaboration) [25]. Disagreements were resolved via discussion and consensus was achieved between the reviewers. As there was no clear consensus quality threshold for study exclusion, papers were not excluded based on quality [26]. The findings of the quality appraisal are shown in Figure 3.

### Data Analysis

Although it was intended to undertake a meta-analysis, this was not possible because of the heterogeneity of the included studies. Therefore, narrative synthesis was used to examine the patterns in the included studies [27]. Similar data from the included studies were identified, analyzed, and reported as themes. These include physical activity interventions, weight loss interventions, and engagement and website use.

## Results

### Study Characteristics

Of the 17 included papers, 2 (12%) papers reported results from the same trial but through different analyses [28,29]; therefore, 16 studies were included in the review (Table 1). Of the 16 studies, 8 (50%) studies were conducted in Australia [29-36], 6 (38%) studies in the United States [37-42], 1 (6%) study in Denmark [43], and 1 (6%) study in Malaysia [44]. Of 16 studies, 9 (56%) assessed the effect of a social media–based intervention only on physical activity [29,31,32,34-37,41,44], and 6 (38%) examined the impact of social media interventions on body weight [30,33,38,40,42,43]. Only 1 (6%) of studies examined the effect of the social media intervention on both physical activity and weight [39].

**Table 1:** Summary of included articles.

Studies based on outcome measures		Country and sample characteristics	Intervention	Outcome measures	Results
**Physical activity**					
	Alley et al [28]	Australia 504 participants from the WALK 2.0 trial (aged ≥18 years)	Paper logbook to record steps, hard copy education material, and pedometer (n=171). Web 1.0: pedometer, website to record steps, public forum, and education material (n=165). Web 2.0: WALK 2.0 website, which provides more social network features for the participant, in addition to the same features in the Web 1.0 group (n=168).	Physical activity (Acti Graph monitors) at 3, 12, and 18 months. Website use (time on site, number of log-ins, and number of step entries) at 3, 12, and 18 months.	The mean difference of MVPA^a^ min/day for Web 2.0 vs log and >55 vs <55 years was 13.7 (range 1.1 to 26.4; *P*<.05) at 3 months. The mean difference of MVPA min/day for Web 1.0 vs log and >55 vs <55 years was 8.3 (range −4.8 to 21.3; *P*>.05) at 3 months. There was a significant difference in time spent on Web 2.0 compared with Web 1.0 for older adults compared with younger adults at (*P*>.05).
	Cavallo et al [37]	United States 134 female undergraduate students (aged <25 years)	Intervention (n=67): INSHAPE^b^ website (web-based education materials and self-monitoring tool) and Facebook group. Control (n=67): web-based education materials and the same news stories related to physical activity provided to the Facebook group delivered through email.	Physical activity (Paffenbarger activity questionnaire) at 12 weeks.	The mean calories used in physical activity was 2248.98 (SD 1541.19) for the control group, compared with 2394.75 (SD 1448) for the intervention group (*P*>.05).
	Chee et al [44]	Malaysia 147 government employees with metabolic syndrome (aged 18-59 years)	Control (n=103): pedometer, physical activity information via pamphlet, log card, and regular meeting. Intervention (n=44): same as control group plus joining a Facebook group.	Physical activity (number of steps per day/accelerometer) at 4 and 6 months.	The mean number of steps per day for the control group were 3938.95 (SD 1276.29), 4459.15 (SD 1282.52), and 4318.06 (SD 1293.11) at baseline, 4 months, and 6 months, respectively (*P*>.01). The mean number of steps per day for the Intervention group were 3897.50 (SD 1188.69), 7192.20 (SD 1925.55), and 6161.30 (SD 1603.97) at baseline, 4 months, and 6 months, respectively (*P*>.01). The mean number of steps per day for the intervention group were 7192.20 (SD 1925.55) and 6161.30 (SD 1603.97) at 4 months and 6 months, respectively, compared with 4459.15 (SD 1282.52) and 4318.06 (SD 1293.11) for the control group (*P*>.01).
	Duncan et al [31]	Australia 301 male (aged 35-54 years)	IT-based intervention (n=205): education materials, self-monitoring, and automated feedback based on progress in physical activity and dietary behavior goals. Ability to interact with other participants. Print-based intervention (n=96): education materials and capacity to self-monitor physical activity and dietary behaviors.	Physical activity (the Active Australia Questionnaire). Dietary behaviors (19 items adapted from existing instruments).	Self-reported physical activity (min/week) at 95% CI exp (β)=1.03 (0.78-1.36) for IT-based vs print-based (*P*>.05). Dietary score at 95% CI exp (β)=0.97 (0.75-1.25) for IT-based vs print-based (*P*>.05). Self-reported physical activity (min/week) at 95% CI were exp (β)=1.45 (1.09-1.95) and exp (β)=1.55 (1.14-2.10) at 3 and 9 months, respectively, in both groups (*P*>.01). Dietary scores at 95% CI were exp (β)=1.07 (1.03-1.11) and exp (β)=1.10 (1.05-1.13) at 3 months and 9 months, respectively, in both groups (*P*>.01).
	Edney et al [32]	Australia 444 adults (aged 18-65 years)	Basic group (n=160): pedometer and access self-monitoring feature of Active Team app. Socially enhanced group (n=141): pedometer and access to a full Active Team app, which includes gamification and social features. Control group (n=143): no intervention.	Physical activity (GENEActiv accelerometers) at 3 and 9 months. Physical activity (the Active Australia Survey) at 3 and 9 months.	The mean difference between groups in MVPA were 0.32 and 0.47 at 3 and 9 months, respectively (*P*>.05). The mean difference between groups in self-reported physical activity was 1.04 (*P*>.05) and 2.62 (*P*>.05) at 3 and 9 months, respectively.
	Kolt et al [29]	Australia 504 insufficiently active adults (aged ≥18 years)	Paper logbook to record steps, hard copy education material, and pedometer (n=171). Web 1.0: pedometer, website to record steps, public forum, and education material (n=165). Web 2.0: WALK 2.0 website which provides more social network features for participants in addition to the same features in the Web 1.0 group (n=168).	Physical activity (Acti Graph monitors) at 3, 12, and 18 months. Website use (time on site, number of log-ins, and number of step entries) at 3, 12, and 18 months.	The mean difference in MVPA for Web 1.0 vs Web 2.0 from baseline at 95% CI were −7.3 (−12.3 to −2.4; *P*<.01), 1.1 (−4.4 to 5.6; *P*>.05), and 2.5 (−4.5 to 9.5; *P*>.05) at 3, 12, and 18 months, respectively. The mean difference in MVPA for Web 1.0 vs logbook from baseline at 95% CI were −5.3 (−9.9 to −0.6; *P*<.05), 0.0 (−6.2 to 6.1; *P*>.05), and 1 (−6.6 to 8.5; *P*>.05) at 3, 12, and 18 months, respectively. The mean difference in MVPA for Web 2.0 vs logbook from baseline at 95% CI were 2.1 (−2.4 to 6.5; *P*>.05), −1.2 (−6.5 to 4.2; *P*>.05), and −1.5 (−7.5 to 4.4; *P*>.05) at 3, 12, and 18 months, respectively. The mean time on site (second/week) for Web 1.0 was 386.40 (SD 371.80), 121.54 (SD 219.39), and 88.99 (SD 214.08) compared with 713.32 (SD 948.75), 305.47 (SD 488.29), and 188.90 (SD 291.74) for Web 2.0 at 3, 12 and 18 months, respectively (*P*<.01).
	Looyestyn et al [34]	Australia 89 inactive adults (aged 18-50 years)	Intervention group (n=41): Facebook group program, included daily interactive posts, and details of the running sessions. Control group (n=48): hard copy of the running program.	Physical activity (Active Australian Survey) at 2 and 5 months.	The mean self-reported MVPA for the intervention group were 409.5 (SE 52.8) and 398.3 (SE 52.8) at 2 months, and 5 months, respectively, compared with 269.0 (SE 47.5) at baseline (*P*<.05). The mean self-reported MVPA for the control group was 450.8 (SE 48.3) and 309.8 (SE 52.1) at 2 months, and 5 months, respectively compared with 359.6 (SE 43.9) at baseline (*P*<.05). The mean difference between group-by-time interaction was 3.39 (*P*<.05).
	Maher et al [35]	Australia 110 adults (aged 18-65 years)	Intervention group (n=51 individuals, 12 teams): Active Team app included self-monitoring and social elements and pedometers. Control group (n=59 individuals, 13 teams): no intervention.	Physical activity (Active Australia Survey) at 8 and 20 weeks.	The mean change of overall physical activity from baseline to 8 weeks for the intervention group was 248 (SE 59) compared with 113 (SE 43) in the control group (*P*<.05). The mean change of overall physical activity from baseline to 20 weeks for the intervention group was 97 (SE 50) compared with 56 (SE 47) in the control group (*P*>.05).
	Rote et al [41]	United States 63 female college students (mean 18.6, SD 0.7 years)	Standard walking intervention (n=31): feedback on baseline physical activity level, a pedometer, and a paper log. Facebook social support group (n=32): same as a standard plus Facebook group.	Physical activity (daily step count/pedometer).	The mean step/day for the Facebook group was 12,472.44 (SD 2816.61) at 8 weeks, compared with 5595.10 (SD 1729.48) at baseline (*P*<.05). The mean step/day for the standard group was 10,135.64 (SD 3316.37) at 8 weeks, compared with 5595.10 (SD 1729.48) at baseline (*P*<.05). The mean step/day for the Facebook group was 12,472.44 (SD 2816.61), compared with 10,135.64 (SD 3316.37) for the standard group at 8 weeks (*P*<.05).
	Vandelanotte et al [36]	Australia 1328 adults signing up for the 10,000 steps program (aged ≥18 years)	Web 1.0 group (n=899): web-based step log, discussion forum, and web-based education materials. Web 2.0 group (n=868): same as Web 1.0 and access to WALK 2.0 website, which provides more social network features for participants.	Physical activity (the Active Australia Survey) at 3 months. Website Engagement (Google Analytics) at 3 months.	The mean physical activity (min/week) was 381.7 (SD 16.6) for Web 1.0 compared with 473.9 (SD 26.4) for Web 2.0 at 3 months (*P*=.05). The mean time on site (second/week) was 195 (SD 464) for web 1.0 compared with 179 (SD 678) for Web 2.0 at 3 months (*P*>.05).
**Weight**					
	Balk-Møller et al [43]	Denmark 566 employees in the social welfare and health care sector (aged ≥18 years)	Intervention group (n=355): access to SoSu-life tool—self-reporting of diet and exercise and feedback, weekly assignment, and colleagues’ challenge. Each participant chose 1 pledge out of 7 to focus on: lose weight, eat healthier, improve physical fitness, improve physical strength, quit smoking, or maintain a healthy lifestyle. A total of 154 (44%) participants and 74 (35%) participants chose weight loss pledges at 16 weeks and 38 weeks, respectively. Control group (n=211): no intervention.	Weight (digital scale) at 16 and 38 weeks. Body fat waist circumference.	The mean difference between the weight loss subgroup and control group in body weight was −2.36 (−3.23 to −1.49; *P*<.01) and −1.64 (−3.04 to −0.24; *P*<.05) at 16 weeks and 38 weeks, respectively. The mean difference between the weight loss subgroup and control group in body fat were −0.99 (−1.63 to −0.34; *P*<.05) and −0.39 (−1.43 to 0.64; *P*>.05) at 16 weeks and 38 weeks, respectively. The mean difference between the weight loss subgroup and control group in waist circumference were −2.45 (−4.09 to −0.81; *P*<.05) and −2.47 (−4.30 to −0.63; *P*<.05) at 16 weeks and 38 weeks, respectively.
	Brindal et al [30]	Australia 2648 adults affected by overweight or obesity (aged ≥18 years)	Information-based (n=53): noninteractive website with dietary information. Supportive (n=1314): dietary information, weight tracker, meal planner, and social networking platform. Personalized-supportive (n=1281): dietary information, weight tracker, meal planner with personalized recommendations, and social networking platform.	Weight (self-reported web-based questionnaire) at 12 weeks. Website use (the total number of days that the site was used, the last day the site was used, and days between the first and last use of the site) at 12 weeks.	Average weight loss among completers was 4.15% (SD 4.26%), 4.22% (SD 4.34%), and 3.97% (SD 3.73%) for information-based, supportive, and personalized-supportive, respectively; *P*>.05 at 12 weeks. The average days the site was used were 3.43 (SD 4.28), 5.50 (SD 10.35) and 5.50 (SD 10.35) for the information-based, supportive, and personalized-supportive websites, respectively (*P*<.05).
	Godino et al [38]	United States 404 college students affected by overweight or obesity (aged 18-35 years)	Intervention group (SMART^c^ intervention; n=202): education materials, self-monitoring and feedback, goal-setting, and challenge through 6 modalities (Facebook, mobile apps, SMS text messaging, emails, a website, and technology-mediated communication with a health coach). Control group (n=202): general health information through a website and email.	Weight (digital scale) at 6, 12, 18, and 24 months.	The mean difference in weight between the intervention group and control group was −1.33 kg and −1.33 kg at 6 months and 12 months, respectively (*P*<.05). The mean difference in weight between the intervention group and control group was −0.67 kg and −0.79 kg at 18 months and 24 months, respectively (*P*>.05).
	Jane et al [33]	Australia 137 adults affected by overweight or obesity (aged 20-65 years)	Facebook group (n=46): weight management program through the Facebook group, and pedometer. Pamphlet group (n=46): weight management program via a booklet and pedometer. Control group (n=45): follow Australian government dietary guidelines and National Physical Activity Guidelines for adults.	Weight (digital scale) at 6, 12, 18, and 24 weeks.	The mean weight loss in the Facebook group were −2.5 (*P*<.05), −3.5 (*P*>.05), −4.9 (*P*<.05), and −4.8 (*P*<.05) compared with −1.1, −1.8, −2.0, and −1.5 for the control group at 6, 12, 18, and 24 weeks, respectively. The mean weight reduction in the pamphlet group was −2.7 (*P*<.05), −3.4 (*P*>.05), −4.5 (*P*<.05), and −3.6 (*P*<.05) compared with −1.1, −1.8, −2.0, and −1.5 for the control group at 6, 12, 18, and 24 weeks, respectively.
	Napolitano et al [40]	United States 52 college students (aged 18-29 years)	Facebook (n=17): handouts and podcasts about diet and activity and access to polls and healthy activity. Facebook Plus (n=18): same as Facebook plus goal-setting, self-monitoring, and social support. Control group (n=17): no intervention.	Weight (scale) at 4 and 8 weeks.	Weight changes were −0.46 (SD 6 1.4) kg, −1.7 (SD 6 1.6) kg, and 0.28 (SD 6 1.7) kg for Facebook, Facebook Plus, and the control groups at 4 weeks, respectively (*P*<.05). Weight changes were −0.63 (SD 2.4) kg, −2.4 (SD 2.5) kg, and −0.24 (SD 2.6) kg for Facebook, Facebook Plus, and the control group at 8 weeks, respectively (*P*<.05).
	Turner-McGrievy, et al [42]	United States 96 adults affected by overweight or obesity (aged 18-60 years)	Podcast only (n=49): 2 podcasts on nutrition and physical activity and a book with calorie information. Podcast + mobile groups (n=47): same as former plus use of a diet and physical activity monitoring app on mobile device and interaction with study counselors and other participants on Twitter.	Weight loss (digital scale).	The mean change in body weight for the podcast group were −2.6 (SD 3.8) and −2.7 (SD 5.1) at 3 months and 6 months, respectively, compared with −2.6 (SD 3.5) and −2.7 (SD 5.6) for podcast +mobile groups (*P*>.05).
**Physical activity and weight**					
	Greene et al [39]	United States 349 adults (aged 18-79 years)	Intervention group (I Well OSNs^d^; n=180): education material on diet and physical activity, access to I Well OSNs and pedometer. Control group (n=169): education material on diet and physical activity.	Physical activity (SQUASH)^e^. Weight (digital scale).	The average of all physical activity (min/week) for the intervention group was 2479.3 and 2686.9 compared with 2102.4 and 2248.2 for the control group at 3 and 6 months, respectively (*P*>.05). Leisure time walking (min/week) for the intervention group was 354.1 and 341.0 compared with 160.4 and 208.6 for the control group at 3 and 6 months, respectively (*P*<.05). The mean weight loss for the intervention group was 4.4 pounds and 5.2 pounds compared with 0.9 pounds and 1.6 pounds for the control group at 3 and 6 months, respectively (*P*<.05).

^a^MVPA: moderate to vigorous physical activity.

^b^INSHAPE: Internet Support for Healthy Associations Promoting Exercise.

^C^SMART: Social Mobile Approaches to Reducing Weight.

^d^OSNs: online social networks.

^e^SQUASH: Short Questionnaire to Assess Health-Enhancing physical activity.

### Intervention Description

The duration of the interventions ranged from 2 months [34,40,41] to 24 months [38]. There was heterogeneity among the included studies (n=16), in terms of both the duration and components of the intervention. Facebook was used as the primary intervention modality in 1 study [34], in which a closed Facebook group delivered the content of a running program. However, Facebook was part of a multicomponent intervention in 38% (6/16) of studies [33,37,38,40,41,44]. Facebook groups were mostly closed or private and were used to deliver intervention material, goal-setting, and feedback and to provide social support and interaction between participants and study coordinators or participants themselves. The Facebook group was led by either a study moderator [33,34,37,41,44] or a health coach [38].

A second-generation website (Web 2.0), which has more interactive social networking features than Web 1.0, was used as an intervention in 25% (4/16) of studies [29,30,36,39]. Overall, 12% (2/16) of studies used both a website and mobile app [31,43]; 12% (2/16) of studies used mobile apps linked to Facebook [32,35], and 6% (1/16) of studies used Twitter in conjunction with a website [42]. Generally, websites or mobile were used to provide intervention materials and self-monitoring, such as logging steps.

Most studies (12/16, 75%) compared social media interventions with other types of interventions. Overall, 44% (7/16) of studies compared social media with educational materials (eg, booklets or pamphlets, podcasts, and web-based education materials) [30,33,34,37-39,42]. Other types of interventions included paper logbooks with a pedometer for self-monitoring [41,44], noninteractive websites [36], or a combination of both [29]. Furthermore, 1 (6%) study used a print-based intervention as a comparator, which consisted of educational material and self-monitoring [31].

### Physical Activity Interventions

Of the 10 (63%) studies that measured physical activity as an outcome, 9 (90%) studies measured physical activity alone [29,31,32,34-37,41,44], and 1 (10%) study measured physical activity and body weight [39]. Physical activity was measured subjectively through self-report in 60% (6/10) of studies [31,34-37,39], whereas 40% (4/10) of studies measured physical activity objectively through either an accelerometer [32,44] or a pedometer [29,41]. One of these studies measured physical activity via self-reporting as a secondary outcome [32].

Although 70% (7/10) of studies reported a significant improvement in physical activity between groups [29,34-36,39,41,44], 30% (3/10) of studies found no significant improvement in physical activity between groups [31,32,37]. Interestingly, 67% (2/3) of the studies that found no significant improvement reported significant physical activity changes over time within groups [31,37]. However, Edney et al [32] found that physical activity improved only at 9 months when measured subjectively and there was no significant effect when measured objectively.

As most of the interventions were multicomponent, it was difficult to conclude which components contributed to the improvements in physical activity. Furthermore, there was inconsistency in the effects of some features on physical activity improvement. For example, gamification was used within the intervention in 2 studies [32,35]. Although Maher et al [35] reported a significant improvement in physical activity, Edney et al [32] reported no significant difference between the groups.

Overall, 30% (3/10) of studies assessed the predictors of physical activity improvement [34,35,41]. These studies reported a significant correlation between baseline physical activity and changes in physical activity (values ranging from *P*<.001 to *P*=.04), that is, the lower the baseline physical activity level, the greater the improvement. In 2 studies, adherence to the intervention was significantly correlated with physical activity improvement. within these studies greater adherence saw more improvement observed in physical activity (*P*=.03-.04) [34,35]. Conversely, Rote et al [41] found that adherence to the intervention was not a predictor of change in physical activity.

### Weight Loss Interventions

A total of 38% (6/16) of studies assessed the effect of a social media intervention on body weight [30,33,38,40,42,43], and 6% (1/16) of studies measured both weight and physical activity [39]. All (5/6, 86%) but 1 study used a digital scale for staff to measure weight. The other study used self-reported weight [30]. Metabolic parameters (BMI, waist circumference, and body fat) were measured as a secondary outcome in 43% (3/7) of studies [33,38,43].

Significant differences between groups regarding weight loss (in favor of the social media intervention group) were reported in 57% (4/7) of studies [33,38,39,43]. Metabolic parameters were found to be substantially improved among the social media intervention group, corresponding with weight reduction [33,38,43]. In contrast, 29% (2/7) of studies did not find significant differences between groups [30,42]. The last study, which had 3 groups (Facebook, Facebook plus, and the control group), found a significant difference in weight loss between the Facebook plus group and the control group. However, there was no significant difference between the control group and the Facebook group, which lacked additional features that were in the “Facebook plus” group (daily SMS text messages, personalized feedback, and “support buddy”) [40].

Different factors could contribute to the inconsistency in the differences between the groups. Although there were social features within the intervention, their use was not consistent. For example, Balk-Møller et al [43] reported that the use of social features was optimal, as the participants knew each other before the study, which could improve the effectiveness of the intervention. However, low use of social features was reported in 3 studies [30,33,42], as participants were reluctant to contact unfamiliar people. However, despite the low use of these social features, it displaced real-time support from family and friends, which eventually decreased the effect of the intervention [30,33,42].

Seasonality is another factor that may have affected the intervention efficacy. Overall, 29% (2/7) of studies were conducted during the Christmas and New Year periods, which reportedly diluted the effect of the intervention [33,42]. Moreover, 29% (2/7) other studies highlighted the effect of intervention dose as a factor that improved intervention efficacy; the greater the use of social media intervention, the greater the efficacy of the intervention [39,42]; 29% (2/7) of studies found that self-monitoring, whether electronic or paper-based, was associated with improved weight loss [30,42].

### Engagement and Website Use

Engagement and use metrics were reported in 69% (11/16) of included studies [29-32,34-38,40]. There were no standard metrics for measuring engagement with various measures used. These include time spent on site; frequency of logging on to the platform; and the number of interactions (eg, likes, comments to other participants, sending messages, or responding to events). High engagement was defined as logging into the website regularly and consistently throughout the study period. Low engagement was considered to occur when there were few or no regular interactions with the website. There was no clear threshold between the definitions of high and low engagement in the studies.

A total of 44% (7/16) of studies reported a decline in engagement throughout the study [29-31,36-38,41]. These studies ranged in duration from 2 months [41] to 24 months [38]. However, 4 (25%) studies, conducted over 2 to 3 months [32,34,35,40], achieved high engagement throughout the intervention. Furthermore, 13% (2/16) of studies reported that gamified features could encourage high engagement [32,35]. Another study suggested that using Facebook mainly to deliver the intervention content rather than being part of the intervention and the diversity of posts on Facebook groups might improve engagement [34].

Studies that compared Web 2.0 (with social functionalities) with Web 1.0 (as a comparison group) found that Web 2.0 had a significantly higher use [30] and step entry [29] compared with Web 1.0. Engagement and use were found to be higher in Web 2.0 group in other studies [31,32,37], but it was unclear if the difference was significant. Vandelanotte et al [36] reported a significant difference between the websites in terms of the number of visits and average number of visits; however, there was no difference in terms of the time spent on the sites.

The relationship between engagement with the intervention and behavior change was unclear. Participant engagement was not a predictor of behavior change in 45% (5/11) of studies [31,32,36,38,41]. However, engagement and use of social media interventions was found to be related to behavior change in 55% (6/11) of studies [30,34,35,37,39,40].

## Discussion

### Social Media Intervention Efficacy

This review found varied results from randomized controlled trials regarding the effect of social media interventions on increasing physical activity or reducing weight in the short term and no evidence of long-term effectiveness. Several factors may have contributed to this finding. First, given the multifaceted nature of the interventions and various combinations of components, it was difficult to elucidate which components contributed to the intervention’s efficacy. Second, although some features were intended to optimize the effect of the intervention (eg, social support), the use of these features was often suboptimal or decreased supportive contact with family and friends. Finally, participants’ engagement in the intervention was variable. The intervention dose in the included studies was not sufficient to improve physical activity or weight loss.

Previous reviews have highlighted that social media can positively impact some aspects of behavior change, such as improved physical activity levels, better choices of healthy food, and weight management; however, the evidence of social media intervention efficacy was inconclusive in these reviews [18,45,46]. In contrast, other reviews and meta-analyses have found that social media interventions do not have a significant effect on behavior change [19,20,47]. This highlights the need for further robust research to provide definitive evidence-based recommendations for clinical practice.

A key omission in the included studies was the involvement of health care professionals. Despite nurses and physicians being trusted by patients to provide health advice and support for behavior change [48], none of the included studies involved health care providers in the interventions. Ventola [49] argues that social media use by health care providers can improve health outcomes and foster communication. Delivery of social media intervention by health care providers can also improve intervention efficacy, as patients use social media to complement health care professionals rather than as a substitute [50]. Future studies of social media interventions should consider how these interventions might seek to close the gap with existing health care services rather than creating a different avenue for preventive care.

### The Relationship Between Engagement and Behavior Change

This review found that engagement with the intervention frequently decreased over time, particularly with longer interventions. This result could be expected, as people tend to be less likely to engage in long-term interventions [51]. This is consistent with previous reviews, which also demonstrated a decrease in engagement over time [46,52]. The decrease in engagement could also be a factor impacting the limited evidence of long-term improvements in health outcomes.

This review found that the evidence regarding engagement as a predictor of behavior change is inconsistent. Previous studies have also reported similar inconsistencies. Although Murray et al [53] found that engagement was not a predictor of behavior change, Hageman et al [54] reported that engagement with some features could predict weight loss, whereas engagement with other features could not. However, because there were disparate metrics for measuring engagement, comparisons are difficult. Furthermore, engagement could be subject to the “lurking” concept. Lurking refers to viewing the intervention components without active interaction [55]. Therefore, these results should be cautiously interpreted. To understand engagement and behavior change, it is necessary to standardize engagement metrics and consider the impacts of passive engagement [31,38].

### Strengths and Limitations

The presence of a transparent protocol, quality appraisal, and involvement of multiple reviewers are the strengths of this review. However, this review has some limitations. First, only 2 databases were used in this study, as they resulted in a large number of studies being identified as potentially appropriate. The use of a single reviewer to screen titles and abstracts may have introduced bias, although they did screen the papers twice and seek advice from coauthors before making a final determination. In addition, inconsistent reporting within the included studies made it challenging to appraise study quality and extract data. Although quality appraisal provides insight into the quality of the included papers, it was not used to exclude studies, given the lack of a consistent definition of a quality cutoff. Finally, the lack of control groups who received “usual” care made it difficult to determine efficacy.

### Conclusions

Despite the promise of social media interventions in supporting behavior change for physical activity and weight loss, the evidence regarding their effectiveness is mixed. More robust research is needed to identify which components or functionality of the social media intervention has the most effect on behavior change and the dose of the intervention that best achieves the effect with a minimal effect on engagement. Furthermore, the relationship between engagement and behavior change needs to be clearly understood.

## Abbreviations

PRISMA: Preferred Reporting Items for Systematic Reviews and Meta-Analyses
